# Supplementary material for: Characterizing cell-type spatial relationships across length scales in spatially resolved omics data
Source: Nat Commun. 2025 Jan 3;16:350. doi: 10.1038/s41467-024-55700-1 (PMC11699133; doi:10.1038/s41467-024-55700-1)
Supplement: Supplementary file 3 — Supplementary Software 1 [file 41467_2024_55700_MOESM3_ESM.zip › CRAWDAD/plots/spleen/S9_spleen_cd4_near_neutro_trends_pbvn.pdf]

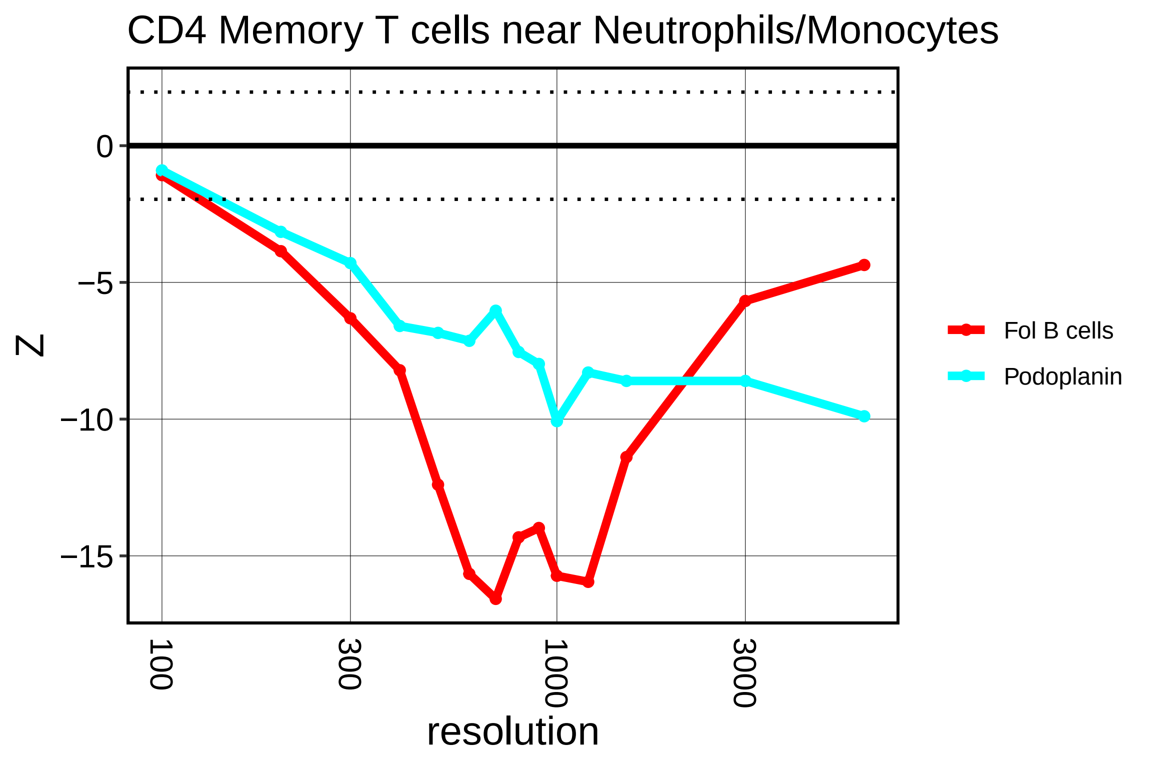
CD4 Memory T cells near Neutrophils/Monocytes
0
−5
−10
−15
Z
100
300
1000
3000
resolution
Fol B cells
Podoplanin
